# Supplementary material for: Nonlinear spectral modeling and control of soft-robotic muscles from data
Source: Nat Commun. 2026 Sep 16;17:10897. doi: 10.1038/s41467-026-77664-0 (PMC13677678; doi:10.1038/s41467-026-77664-0)
Supplement: Supplementary file 1 [file 41467_2026_77664_MOESM1_ESM.pdf]

Nonlinear Spectral Modeling and Control of Soft-Robotic Muscles from Data

Supplementary Material

Leonardo Bettini[1†], Amirhossein Kazemipour[2†], Robert K. Katzschmann[2*], George Haller[1*]

[1]Institute for Mechanical Systems, ETH Zurich, Leonhardstrasse 21, 8092 Zurich, Switzerland.
[2]Soft Robotics Lab, ETH Zurich, Tannenstrasse 3, 8092 Zurich, Switzerland.

*Corresponding author(s). E-mail(s): rkk@ethz.ch; georgehaller@ethz.ch;
†These authors contributed equally to this work.

1 General justification for additive slow forcing

Let us consider the non-autonomous dynamical system

$$\begin{cases} \dot{\boldsymbol{x}} = \boldsymbol{F}(\boldsymbol{x}, \boldsymbol{u}) & \boldsymbol{F}, \in \mathcal{C}^r, \\ \dot{\boldsymbol{u}} = \epsilon \boldsymbol{w}(\boldsymbol{x}, \epsilon t), & 0 \leq \epsilon \ll 1, \end{cases} \tag{1}$$

where $\boldsymbol{x} \in \mathbb{R}^n$ and $\boldsymbol{u}(t) \in \mathbb{R}^f$ denotes a time-dependent external forcing. We use the notation

$$\boldsymbol{F}(\boldsymbol{x}, \boldsymbol{0}) = \tilde{\boldsymbol{f}}_0(\boldsymbol{x}). \tag{2}$$

Consider now the integral

$$\int_0^1 \frac{d}{ds} \boldsymbol{F}(\boldsymbol{x}, (1-s)\boldsymbol{u})\, ds, \tag{3}$$

which can be evaluated as

$$\int_0^1 \frac{d}{ds} \boldsymbol{F}(\boldsymbol{x}, (1-s)\boldsymbol{u})\, ds = \tilde{\boldsymbol{f}}_0(\boldsymbol{x}) - \boldsymbol{F}(\boldsymbol{x}, \boldsymbol{u}). \tag{4}$$

On the other hand, by the chain rule, we have

$$\int_0^1 \frac{d}{ds} \boldsymbol{F}(\boldsymbol{x}, (1-s)\boldsymbol{u})\, ds = -\left[\int_0^1 \boldsymbol{D_u F}(\boldsymbol{x}, (1-s)\boldsymbol{u})\, ds\right] \boldsymbol{u}, \tag{5}$$

where $\boldsymbol{D_u F}$ denotes the Jacobian of $\boldsymbol{F}$ with respect to the input $\boldsymbol{u}$. Equating (4) and (5) yields the decomposition

$$\boldsymbol{F}(\boldsymbol{x}, \boldsymbol{u}) = \tilde{\boldsymbol{f}}_0(\boldsymbol{x}) + \boldsymbol{f}_1(\boldsymbol{x}, \boldsymbol{u}), \tag{6}$$

where

$$\boldsymbol{f}_1(\boldsymbol{x}, \boldsymbol{u}) \doteq \left[\int_0^1 \boldsymbol{D_u F}(\boldsymbol{x}, (1-s)\boldsymbol{u})\, ds\right] \boldsymbol{u}. \tag{7}$$

By further splitting the autonomous part into its linear and nonlinear components,

$$\tilde{\boldsymbol{f}}_0(\boldsymbol{x}) = \boldsymbol{A}\boldsymbol{x} + \boldsymbol{f}_0(\boldsymbol{x}), \tag{8}$$

we recover the form of Eq. (4) in the main text.

2 Procedure for the parametrization of the adiabatic Spectral Submanifold and its reduced dynamics from data

We elaborate here on Section 4.1 (Methods) of the main manuscript, focusing on the data-driven model reduction approach based on the adiabatic Spectral Submanifold (aSSM). Our goal is to obtain a leading-order approximation of the aSSM, $\mathcal{M}_\epsilon$. Despite allowing a nonzero ϵ in Eq. (4) of the main manuscript, we approximate both the aSSM, $\mathcal{M}_\epsilon$, and its associated slow manifold (SM), $\mathcal{L}_\epsilon$, with their frozen-limit counterparts $\mathcal{M}_0$ and $\mathcal{L}_0$, which provide close approximations for $\epsilon > 0$ small by the smooth dependence of $\mathcal{M}_\epsilon$ and $\mathcal{L}_\epsilon$ on ϵ (see Haller and Kaundinya [1]).

To parametrize the aSSM under this approximation, we generate a family of decaying trajectories by perturbing the system around steady states associated with different constant voltage inputs. These perturbations are introduced by applying abrupt voltage jumps relative to the base constant input. Specifically, after allowing the system to settle into a steady state under a fixed voltage, we suddenly change the input to a new constant value. This abrupt change shifts the steady state to a new point in the extended phase space of the $\epsilon = 0$ system along the external input u axis, effectively turning the previous steady state into a new initial condition. For each voltage value $u = \bar{u}$, we obtain one or more decaying trajectories, which allow us to extract the local parametrization of the Spectral Submanifold (SSM), $\mathcal{W}(E(\bar{u}))$, and its associated reduced dynamics, as detailed in the Methods 4.1 of the manuscript. As illustrated in Supplementary Fig. 1a, this procedure is repeated across a range of voltage values, thereby sampling different slices of the extended phase space. The local SSM parametrizations and their corresponding reduced dynamics, obtained at various voltage levels, are then interpolated to construct a global approximation of the aSSM.

However, the procedure of inducing abrupt voltage jumps to generate initial conditions for decaying trajectories introduces hysteretic effects that are not observed in the normal operational regime of the actuator. This behavior presents a challenge for the practical construction of the aSSM. As seen in Supplementary Fig. 1b, trajectories initiated from different initial conditions but corresponding to the same reference voltage plane $u = \bar{u}$ in the extended phase space may converge to distinct fixed points. While modeling such behavior becomes relevant in regimes involving

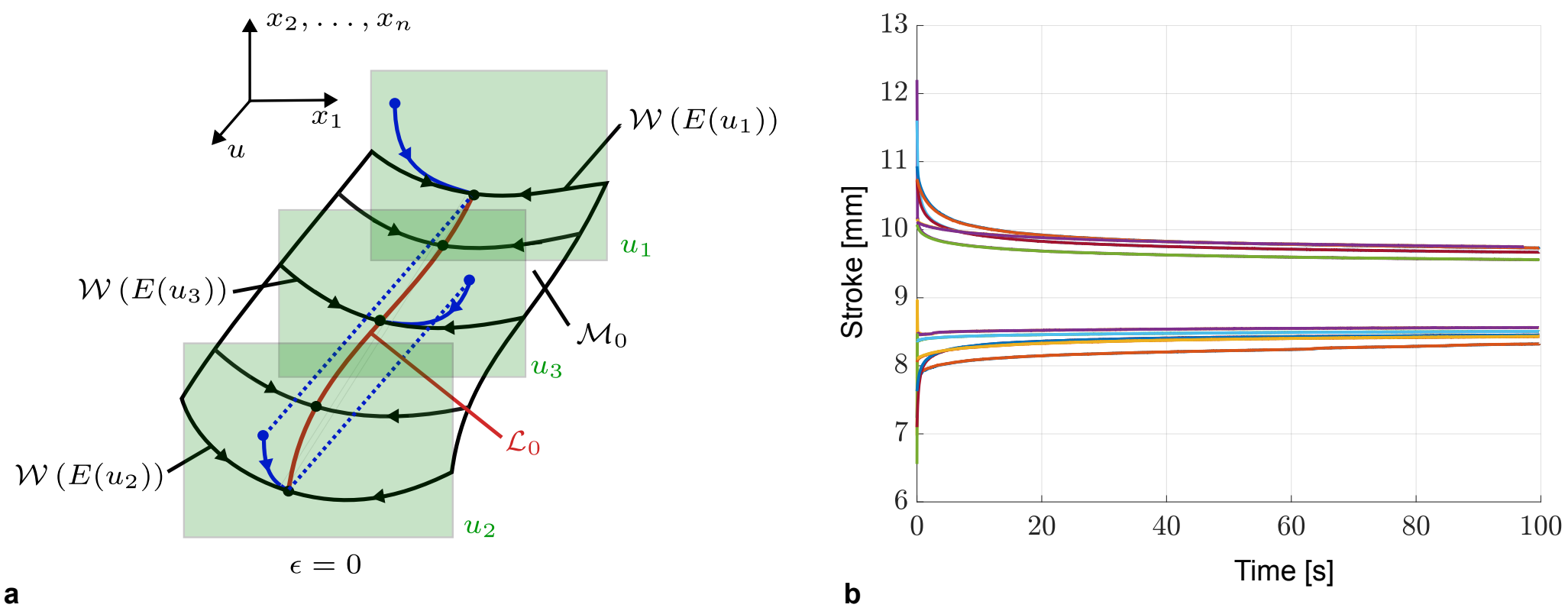

Supplementary Figure 1: (**a**) Procedure for the data-driven leading-order parametrization of the aSSM $\mathcal{M}_0$ and the identification of its reduced dynamics: decaying trajectories (blue) are initiated at constant voltage levels u_1, u_2, u_3 (green planes) and converge to the spectral submanifolds $\mathcal{W}(E(u_i))$ identified at those same voltage levels; the red curve indicates the critical manifold $\mathcal{L}_0$. (**b**) The hysteresis induced by the voltage jumps required to produce distinct initial conditions at a fixed voltage level $\bar{u}$ results in convergence to different fixed points. Here, we report the actuator driven around a baseline voltage of 5 kV with voltage jumps of $\pm[1.5, 2]$ kV. Each colored curve corresponds to an individual trial.

extreme voltage changes, it lies beyond the scope of the present work. In our application, these large input jumps are solely employed to populate the data required for aSSM construction and do not reflect standard actuator usage.

Nevertheless, to consistently define the reduced dynamics on the aSSM, it is necessary to associate a unique fixed point with each voltage value $\bar{u}$. To this end, we calibrate the fixed point selection using the Slow Manifold (SM) obtained from randomly actuated trajectories.

3 Slow Manifold modeling of antagonistic musculoskeletal joint

The antagonistic musculoskeletal joint described in Section 2.4 of the main manuscript employs HASEL actuators in an antagonistic configuration, each coupled with electrostatic clutches, to mimic the actuation of biological limbs (Kazemipour et al. [2]). As discussed in the main text, systems of this type exhibit rapid convergence to the SM, which justifies the use of SM-based modeling to simplify the aSSM-based modeling approach. This simplification involves a direct identification of the SM from forced response data as a mapping from input to observable. Specifically, we consider a scalar input $u(t) \in \mathbb{R}$ and a scalar observable $s(t) \in \mathbb{R}$.

A practical and readily interpretable approach is to approximate this mapping using a polynomial in u, which is also easily invertible, an advantage for control applications requiring inverse mappings. As an alternative, we have also tested radial basis function (RBF) approximation (Powell [3]) with $M = 15$ centers uniformly distributed in the range of the external inputs $[u_{\min}, u_{\max}]$ and variance $\sigma = (u_{\max} - u_{\min})/M$. While still interpretable, RBF models can pose challenges in enforcing monotonicity, making their inversion less straightforward.

We further considered two neural network (NN) architectures: (i) a feedforward neural network (FNN) with one hidden layer of 10 neurons and Tanh activation function (Hagan et al. [4]); (ii) a radial basis function neural network (RBF-NN) with a Gaussian activation first layer and linear output layer (Park and Sandberg [5]). While these models achieve similar predictions and normalized mean trajectory errors (NMTEs) as the polynomial and the RBF models (see Supplementary Fig. 2), they are less interpretable and more difficult to invert.

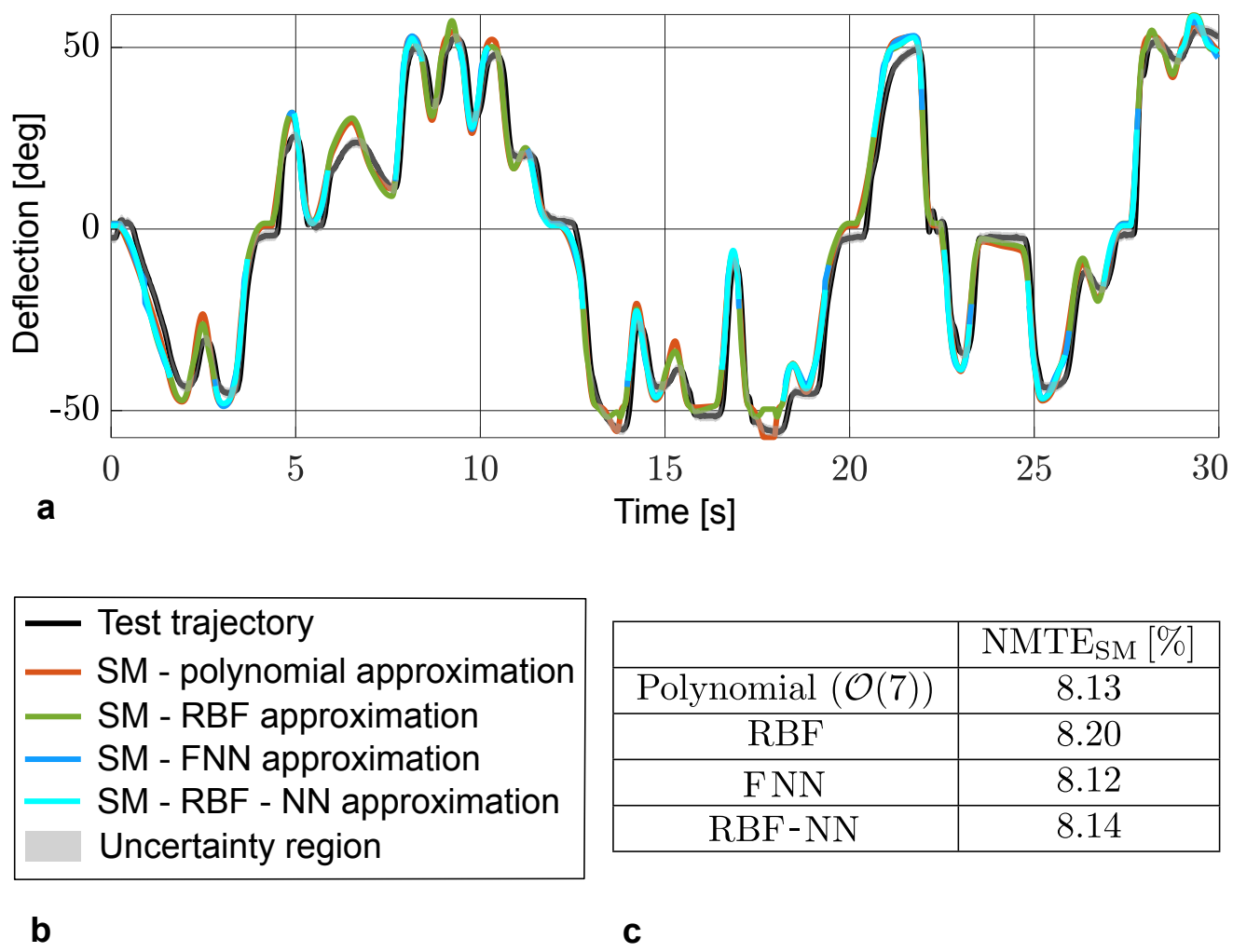

	$NMTE_{SM}$ [%]
Polynomial ($\mathcal{O}(7)$)	8.13
RBF	8.20
FNN	8.12
RBF-NN	8.14

Supplementary Figure 2: Comparison of different data-driven methods for the one-dimensional SM-based modeling of the antagonistic musculoskeletal system: polynomial, radial basis function (RBF), feedforward neural network (FNN), and RBF neural network (RBF-NN) approximations of the slow manifold. (**a**) Time series prediction over a test trajectory. (**b**) Legend. (**c**) Normalized Mean Trajectory Errors (NMTEs).

4 Additional control repeatability and trajectory-generalization analysis

We analyzed repeated control data collected on the same physical antagonistic HASEL–clutch setup to assess repeatability of the learned-map-based controller beyond the representative trial of Fig. 8 of the main text. The original Fig. 8 benchmark trajectory remains the complete three-controller comparison. On this benchmark trajectory, five trials of the combined inverse-SSM+PI controller, denoted FF+PI, including the Fig. 8 configuration and an additional gain-sensitivity trial using the PI-only gain setting, remained in a narrow low-error range. We also analyzed a second random trajectory with a larger target range and higher reference total-variation rate, defined as $T^{-1}\int_0^T |\dot{\theta}_d(t)|\,dt$, where T is the trajectory duration and $\theta_d(t)$ is the desired joint-angle trajectory. For sampled data at times t_i, this quantity was computed as $T^{-1}\sum_i |\theta_d(t_{i+1}) - \theta_d(t_i)|$. On this second random trajectory, FF+PI was compared with FF-only. We also evaluated a sinusoidal trajectory, where repeated FF+PI trials tested performance on a distinct smooth reference class. Supplementary Fig. 3 and Supplementary Table 1 report RMS error and 95th-percentile absolute error because isolated clutch-switching transients can dominate maximum absolute error without representing sustained tracking performance.

Supplementary Table 1: Tracking metrics for the additional repeatability and trajectory-generalization analysis. Values are mean ± standard deviation for repeated trials.

Trajectory	Controller	n	RMS error (°)	95th-percentile $\lvert e\rvert$ (°)
Fig. 8 benchmark	PI-only	1	7.63	15.15
Fig. 8 benchmark	FF-only	1	3.63	6.64
Fig. 8 benchmark	FF+PI	5	2.35 ± 0.18	4.21 ± 0.33
Second random	FF-only	1	6.50	8.81
Second random	FF+PI	2	5.36 ± 0.27	5.90 ± 0.47
Sinusoidal	FF+PI	4	2.42 ± 0.35	4.37 ± 0.70

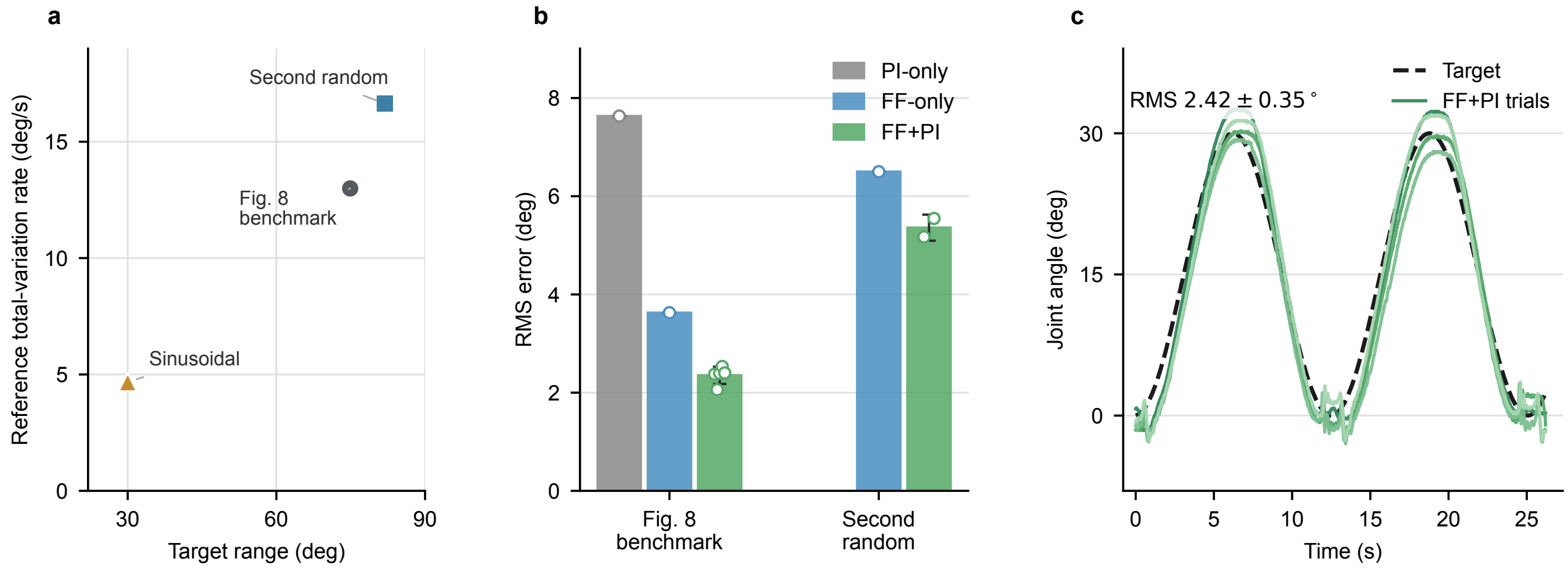

Supplementary Figure 3: Repeatability and trajectory-generalization analysis. (**a**) Target range and reference total-variation rate for the Fig. 8 benchmark, second random, and sinusoidal trajectories. (**b**) Randomized-trajectory comparisons using root-mean-square (RMS) tracking error for the proportional–integral-only (PI-only), feedforward-only (FF-only), and combined feedforward plus feedback (FF+PI) controllers. Bars show mean values, error bars show standard deviation for repeated trials, and circles show individual trials. (**c**) Repeated FF+PI tracking on the sinusoidal trajectory, showing consistent performance of the learned-map-based controller on a distinct smooth reference class. The corresponding 95th-percentile absolute errors are reported in Supplementary Table 1.

5 Analytical modeling of HASEL actuator

We consider the HASEL actuator model presented in Section 2.3 of the main manuscript. In this appendix, we derive the governing equations for a single pouch of the actuator based on the geometric configuration described in Kellaris et al. [6]. Our derivation follows a Lagrangian formalism, which we show to be equivalent to the port-Hamiltonian formalism (van der Schaft and Jeltsema [7], Lohmayer et al. [8]) employed by Yeh et al. [9].

With reference to Fig. 5(a) of the main manuscript, the arc length of the unzipped portion of the pouch is given by

$$l_p(\alpha) = \left(\frac{2A\alpha^2}{\alpha - \sin\alpha\cos\alpha} \right)^{1/2}, \tag{9}$$

while the zipped length is

$$l_e(\alpha) = L_p - l_p(\alpha). \tag{10}$$

The stroke of the actuator, i.e., the contraction from its initial length, is related to α through

$$x = h - \left(l_p(\alpha)\frac{\sin\alpha}{\alpha} + l_e(\alpha) \right), \tag{11}$$

which describes the geometric nonlinearity. The capacitance of the zipped region is given by

$$C(\alpha) = \frac{\epsilon_r \epsilon_0 w}{2t}, l_e(\alpha), \tag{12}$$

where ϵ_r denotes the relative permittivity of the dielectric film and ϵ_0 is the vacuum permittivity. Using Eq. (11), the capacitance can further be expressed as a function of the actuator stroke x.

The single pouch is modeled as a single-degree-of-freedom nonlinear oscillator coupled with an electric circuit. The Lagrangian for the i-th pouch is defined as

$$\mathcal{L}_i = T_i - V_i = \frac{1}{2}m\dot{x}_i^2 - \frac{1}{2}kx_i^2 - mgx_i - \frac{1}{2}\frac{Q_i^2}{C\left(x_i\right)}, \qquad i = 1, \dots, N, \tag{13}$$

where x denotes the vertical stroke, Q is the electric charge accumulated on the electrodes and $C(x_i)$ is the capacitance, which indirectly depends on α through the zipped region. The potential energy includes contributions from the elastic spring, gravity, and the electrostatic energy stored in the capacitor.

To account for dissipative effects, we introduce a Rayleigh dissipation function linear in the vertical velocity, i.e.,

$$\mathcal{R}_i = \frac{1}{2}c\dot{x}_i^2, \qquad i = 1, \dots, N, \tag{14}$$

where c denotes the viscous damping coefficient. Hereafter, we omit the subscript i for simplicity.

The equations of motion, given in Eq. (13) of the main manuscript, are then obtained as

$$\frac{d}{dt}\left(\frac{\partial \mathcal{L}}{\partial \dot{x}}\right) - \frac{\partial \mathcal{L}}{\partial x} + \frac{\partial R}{\partial \dot{x}} = 0. \tag{15}$$

To complete the coupled electromechanical model, we add the governing equation for the electric charge Q accumulating in the zipped region of the electrodes. This is described by the RC-circuit model

$$\dot{Q} = -\frac{1}{RC}Q + \frac{1}{R}u. \tag{16}$$

driven by an external voltage source u. In the literature, the port-Hamiltonian formalism is frequently employed to describe electromechanical systems. Although the term *Hamiltonian* traditionally refers to conservative systems, the port-Hamiltonian approach extends this formalism to include dissipative effects. In this section, we clarify the connection between the dissipative Lagrangian and the port-Hamiltonian formulations by explicitly deriving the equations in a dissipatively perturbed Hamiltonian form.

We define the generalized momentum as

$$p = \frac{\partial \mathcal{L}}{\partial \dot{x}}, \tag{17}$$

which gives

$$\dot{p} = \frac{d}{dt}\left(\frac{\partial \mathcal{L}}{\partial \dot{x}}\right). \tag{18}$$

From Eq. (15), we obtain

$$\dot{p} = \frac{\partial \mathcal{L}}{\partial x} - \frac{\partial \mathcal{R}}{\partial \dot{x}}. \tag{19}$$

We now apply the classic Legendre transformation to transition from the Lagrangian to the Hamiltonian setting. This is achieved by expressing $\dot{x}$ as a function of (x, p), i.e., $\dot{x} = \dot{x}(x, p)$, and defining the Hamiltonian function as

$$\mathcal{H}(x, p) = p\dot{x}(x, p) - \mathcal{L}(x, \dot{x}(x, p)). \tag{20}$$

From this definition, we obtain

$$\frac{\partial \mathcal{H}}{\partial x} = -\frac{\partial \mathcal{L}}{\partial x}, \quad \frac{\partial \mathcal{H}}{\partial p} = \dot{x}. \tag{21}$$

Substituting these expressions into Eq. (19), we obtain the governing equation for the generalized momentum as

$$\dot{p} = -\frac{\partial \mathcal{H}}{\partial x} - \frac{\partial \mathcal{R}}{\partial \dot{x}}. \tag{22}$$

Finally, the coupled evolution equations in a perturbed Hamiltonian form read

$$\begin{aligned} \dot{x} &= \tfrac{\partial \mathcal{H}}{\partial p}, \\ \dot{p} &= -\tfrac{\partial \mathcal{H}}{\partial x} - \tfrac{\partial \mathcal{R}}{\partial p}\tfrac{\partial p}{\partial \dot{x}}. \end{aligned} \tag{23}$$

References

[1] Haller, G., Kaundinya, R.S.: Nonlinear Model Reduction to Temporally Aperiodic Spectral Submanifolds. Chaos **34**(4) (2024) https://doi.org/10.1063/5.0187080

[2] Kazemipour, A., Hinchet, R., Katzschmann, R.K.: Stretchable electrohydraulic artificial muscle for full motion ranges in musculoskeletal antagonistic joints. arXiv preprint arXiv:2409.11017 (2024) https://doi.org/10.48550/arXiv.2409.11017

[3] Powell, M.J.D.: In: Light, W. (ed.) The Theory of Radial Basis Function Approximation in 1990. Oxford University Press, online edn (1992)

[4] Hagan, M.T., Demuth, H.B., Beale, M.H., De Jesús, O.: Neural Network Design. Martin Hagan, Boston (2014)

[5] Park, J., Sandberg, I.W.: Universal approximation using radial-basis-function networks. Neural Comput **3**(2), 246–257 (1991)

[6] Kellaris, N., Venkata, V., Rothemund, P., Keplinger, C.: An analytical model for the design of peano-hasel actuators with drastically improved performance. Extreme Mechanics Letters **29**, 100449 (2019) https://doi.org/10.1016/j.eml.2019.100449

[7] Schaft, A., Jeltsema, D.: Port-hamiltonian systems theory: An introductory overview. Foundations and Trends in Systems and Control **1**(2-3), 173–378 (2014)

[8] Lohmayer, M., Kotyczka, P., Leyendecker, S.: Exergetic port-hamiltonian systems: modelling basics. Mathematical and Computer Modelling of Dynamical Systems **27**(1), 489–521 (2021)

[9] Yeh, Y., Cisneros, N., Wu, Y., Rabenorosoa, K., Le Gorrec, Y.: Modeling and position control of the hasel actuator via port-hamiltonian approach. IEEE Robotics and Automation Letters **7**, 1–8 (2022) https://doi.org/10.1109/LRA.2022.3181365
