# Supplementary material for: Nonlinear spectral modeling and control of soft-robotic muscles from data
Source: Nat Commun. 2026 Sep 16;17:10897. doi: 10.1038/s41467-026-77664-0 (PMC13677678; doi:10.1038/s41467-026-77664-0)
Supplement: Supplementary file 2 [file 41467_2026_77664_MOESM2_ESM.pdf]

Description of Additional Supplementary Files

Supplementary Videos 1-4:

Supplementary Video 1 | Method overview

Supplementary Video 2 | Antagonistic muscle-clutch experimental setup

Supplementary Video 3 | SSM model prediction versus experimental response

Supplementary Video 4 | SSM-enabled model-based control results
