# Supplementary material for: Nonlinear spectral modeling and control of soft-robotic muscles from data
Source: Nat Commun. 2026 Sep 16;17:10897. doi: 10.1038/s41467-026-77664-0 (PMC13677678; doi:10.1038/s41467-026-77664-0)
Supplement: Supplementary file 3 [file 41467_2026_77664_MOESM7_ESM.pdf]

nature portfolio

Peer Review File

Nonlinear Spectral Modeling and Control of Soft-Robotic Muscles from Data

Corresponding Author: Professor George Haller

This file contains all reviewer reports in order by version, followed by all author rebuttals in order by version.

Version 0:

Reviewer comments:

Reviewer #1

(Remarks to the Author)
This manuscript presents a reduced-order data-driven modeling approach for modeling the motion of an antagonistic arm equipped with multiple actuators. The proposed method, called slow manifold, exploits the time scale difference between the fast internal and slow external dynamics of a soft robot to decouple them. This method can be trained using only a few trajectories and achieves an error of a single digit. Finally, the trained model is coupled with a PI controller, and its performance is compared to naive PI and feedforward controllers, demonstrating improvements.

I believe the paper presents interesting results. However, I have a few questions that I think could enhance the paper and improve its readability for the audience.

* Introduction: First two paragraphs missing a lot of references. There are recent reviews on soft robotics which authors could use to back these claims and help navigate the reader. This is true for the discussion as well. Citations to relevant literature are warranted.
* I am confused on why the paragraph starting in Line 256 is not under section 2.3. Since it discusses results for the analytical HASEL model?
* Line 271: Is the antagonistic arm setup similar to that described by Kazemipour et al. [26], or are there any differences? The sentence in line 271 appears to be inspired, and I'm curious if it's been further developed. Could you please explain the modifications made to the setup?
* Why do authors not show the prediction performance of aSSM for antagonistic muscles in Figure 6d? Authors say in paragraph starting in line 279 that the hysteric behavior of actuators makes it challenging to use aSSM. Why is hysteresis not a problem for a single HASEL actuator and becomes challenging for the antagonistic case? Can they clarify this point?
* Figure 6d - Are there any uncertainty regions?
* Figure 8a - x-label is missing
* Can SM make predictions for angles outside the range tested in this paper? For instance, in Figure 8a, the maximum angle tested is approximately 40 degrees. However, Supplementary Movie 2 demonstrates that the arm can reach angles of up to 90 degrees. Does SM have the capability to extrapolate to these values, or is retraining necessary? Is there a specific reason why large angles, such as those not tested, have not been explored?
* I believe the rho number, which defines the dimensionless measure of slowness, is a useful metric that authors define. I'm curious to know the rho values for the tests in Figures 6 and 8. If possible, could they provide the rho values for all the experiments presented in this paper?
* Methods, equation 27: what is S_{i}? Can you define it before and after the equation?
* How do authors address the hysteresis of the artificial muscles in their control algorithm and modeling? Are these effects negligible for the conditions considered in this paper? Or are they not applicable to the HASEL robots, despite being mentioned in the abstract? Alternatively, are these effects time-scales that are much faster than the control frequency?
* SM Figure 1 presents the experimental data, and I'm curious to know the voltage levels involved. Are these voltages pertinent to the operating condition of the antagonistic arm? Additionally, is there any hysteresis observed within the operational voltage range?
* Supplementary movie 3 appears to be presented at a much faster pace compared to real time. I kindly request reviewers to indicate this in the movie. Similarly, supplementary movie 2 could also benefit from the authors marking whether it is presented in real time or not.

(Remarks on code availability)
I was unsuccessful running the code and got the following errors. Maybe the authors can provide more details in the README.

- While running SDOF_SM.m and HASEL_actuator.m
"Unrecognized function or variable 'embedCoordinates'."

- While running antagonistic_joint.m I got the following error "Unrecognized function or variable 'finiteTimeDifference'."

-Also while running HASEL_simulation.m , I got symbolic toolbox error. I suggest authors to mention which MATLAB toolboxes are required to run the examples.

Reviewer #2

(Remarks to the Author)
What are the noteworthy results?

This paper presents a data-driven reduced-order modeling and control strategy for soft artificial muscles, grounded in spectral submanifold (SSM) theory. The authors exploit the pronounced time-scale separation in Hydraulically Amplified Self-Healing Electrostatic (HASEL) actuators to learn a low-dimensional slow manifold (SM) directly from forced-response data. The learned SM provides an explicit input-to-output map that can be inverted in real time for feedforward control. The authors validate this approach on both analytic models and experimental HASEL actuators, and then demonstrate closed-loop control of an antagonistic HASEL–clutch musculoskeletal joint. The combined inverse-SSM feedforward + PI feedback controller achieves substantially lower tracking errors (RMS 2.38°) compared to feedback-only (RMS 7.63°) and feedforward-only (RMS 3.63°) baselines.

Will the work be of significance to the field and related fields? How does it compare to the established literature? If the work is not original, please provide relevant references.

Applying data-driven model reduction to soft robotic control is a significant contribution. The modeling and control of soft artificial muscles has been a persistent challenge due to nonlinearities, hysteresis, and multi-physics coupling. Prior work on SSM-based control has been demonstrated in simulation and on continuum soft robot, but this paper represents an experimental demonstration on antagonistic artificial muscle systems with quantified performance improvements over standard baselines. The distinction between aSSM and SM approaches is well motivated: the SM-based method avoids decay experiments that can trigger hysteresis in HASEL actuators, which is a practical advantage over the aSSM construction. The authors appropriately compare to existing quasi-static models, frequency-domain identification, and Port-Hamiltonian approaches, clearly positioning their contribution as a data-driven alternative that avoids detailed physical parameter identification.

Does the work support the conclusions and claims, or is additional evidence needed?

The authors claim that their SM-based model is "sample-efficient," "predictive," and "computationally light," and that the combined feedforward+feedback controller achieves substantially lower tracking errors. The experimental results in Figures 6 and 8 generally support these claims. The SM model trained on seven forced trajectories achieves single-digit NMTE on both single-actuator and antagonistic-muscle tests, supporting the sample-efficiency claim. The control comparison in Figure 8 clearly demonstrates the performance advantage of the combined controller over the baselines.

However, the claim that this approach is broadly applicable to "other soft robotic systems that exhibit pronounced time-scale separation" (e.g., PAMs, DEAs, SMA actuators) is not supported by any evidence beyond HASEL actuators. This is a speculative statement and should either be toned down or supported with at least a brief numerical demonstration on a different actuator class.

The paper claims in the abstract that the approach "avoids decay experiments that can trigger hysteresis," but the aSSM model (which does use decay data) is also presented extensively throughout the results. The narrative would benefit from a clearer, earlier distinction between the aSSM and SM workflows and when each is appropriate, so that the reader does not come away confused about whether decay data is needed or not.

Are there any flaws in the data analysis, interpretation and conclusions? Do these prohibit publication or require revision?

I have not found significant errors in the data analysis or interpretation. The NMTE metric is well-defined and consistently applied. The control comparison is conducted under identical safety constraints and clutch scheduling, which is a fair benchmarking protocol. One concern is that the PI gains were tuned separately for the combined and feedback-only controllers. While the authors justify this for fairness and stability, it makes it difficult to isolate the contribution of the feedforward term. A supplementary experiment using the same PI gains in both the feedback-only and combined conditions would strengthen the comparison.

Is the methodology sound? Does the work meet the expected standards in your field?

The methodology is generally sound and well-grounded in existing SSM theory. The progression from analytic models to experimental single actuators to the full antagonistic joint is logical and builds confidence in the approach. I have two suggestions regarding the experimental validation:

The robustness of the SM model to environmental changes (e.g., temperature variation, actuator aging, payload changes) is not explored. It would be helpful if the authors could comment on or test how the learned polynomial map degrades over time or under different operating conditions, and how frequently re-calibration might be needed.

The control experiments are demonstrated on a single randomized test trajectory. Repeating the comparison across multiple distinct test trajectories with varying frequency content and amplitude ranges would provide more convincing statistical evidence of the performance gains.

Is there enough detail provided in the methods for the work to be reproduced?

Yes, the authors have provided sufficient detail in the Methods section, and the code and data are made available through an external repository. The step-by-step procedure for data-driven aSSM construction (Methods 4.1) and the SM polynomial fitting (Methods 4.2) are clearly described. The control implementation details, including the anti-windup scheme, clutch scheduling state machine, and safety constraints, are thorough. I am confident that the modeling and control pipeline could be reproduced from the provided information.

Additional Comments

The slowness metric ρ defined in Eq. (7) is central to the validity of the SM approximation, yet the paper does not provide clear practical guidance on what threshold of ρ is acceptable for reliable SM-based control. The analytic examples show that $\rho = 0.1$ works well and $\rho = 1.2$ does not, but the experimental HASEL system operates at $\rho = 0.28$. A more systematic study or clearer guidelines on the acceptable range of ρ would make the method more accessible to practitioners.

The assumption that only one side of the antagonistic muscle is active at a time (mutually exclusive clutch engagement) simplifies the input to a single scalar. The authors acknowledge this as a limitation but do not discuss how significantly this restricts the operational envelope of the joint. It would be useful to discuss in what practical scenarios bilateral co-contraction (simultaneous engagement) would be needed and how much performance is sacrificed by excluding it.

In Figure 6d, the SM prediction of joint deflection shows noticeable deviations at the peaks and sign transitions of the trajectory. It would be helpful if the authors could discuss whether these errors are due to the clutch switching transients, hysteresis effects, or limitations of the polynomial approximation order.

The paper would benefit from a brief discussion of failure modes. Under what conditions would the SM-based controller produce unsafe or unstable behavior? For instance, what happens if the actuator experiences dielectric breakdown or if the clutch fails to engage?

(Remarks on code availability)

Version 1:

Reviewer comments:

Reviewer #1

(Remarks to the Author)
I thank the authors for answering my questions and revising the manuscript. I have no further questions.

(Remarks on code availability)
The authors have updated the README file and provided GitHub links for code dependencies, as well as installation instructions.

Reviewer #2

(Remarks to the Author)
All previous comments/suggestions have been sufficiently addressed in the revised manuscript.

(Remarks on code availability)

Open Access This Peer Review File is licensed under a Creative Commons Attribution 4.0 International License, which permits use, sharing, adaptation, distribution and reproduction in any medium or format, as long as you give appropriate credit to the original author(s) and the source, provide a link to the Creative Commons license, and indicate if changes were made.
In cases where reviewers are anonymous, credit should be given to 'Anonymous Referee' and the source.
The images or other third party material in this Peer Review File are included in the article's Creative Commons license, unless indicated otherwise in a credit line to the material. If material is not included in the article's Creative Commons license and your intended use is not permitted by statutory regulation or exceeds the permitted use, you will need to obtain permission directly from the copyright holder.
To view a copy of this license, visit https://creativecommons.org/licenses/by/4.0/

Response to Reviewer #1

Leonardo Bettini[1†], Amirhossein Kazemipour[2†], Robert K. Katzschmann[2] and George Haller[1]

[1]Institute for Mechanical Systems, ETH Zurich, Leonhardstrasse 21, 8092 Zurich, Switzerland
[2]Soft Robotics Lab, ETH Zurich, Tannenstrasse 3, 8092 Zurich, Switzerland
† These authors contributed equally to this work

June 9, 2026

We thank Reviewer #1 for their positive comments and valuable suggestions. Below, we provide detailed responses to each of the points raised, including excerpts from the revised manuscript with the corresponding changes highlighted in blue.

(1) **Introduction: First two paragraphs missing a lot of references. There are recent reviews on soft robotics which authors could use to back these claims and help navigate the reader. This is true for the discussion as well. Citations to relevant literature are warranted.**

We thank the Reviewer for this suggestion. We have added recent and canonical soft-robotics review citations to the first two paragraphs of the Introduction to support the broad claims about soft-robotic compliance and safe human interaction, wearable and medical applications, artificial muscles, and the modeling and control challenges posed by the nonlinear, hysteretic dynamics of soft actuators. We have likewise added review citations in the Discussion, where the manuscript generalizes from HASEL actuators to broader soft-robotic systems.

We added new references in the first Introduction paragraph as follows:

> *Soft-material-based robots extend these advantages, offering flexibility, adaptability, and compatibility with complex geometries, particularly valuable in human-interactive applications such as surgical tools and prosthetics (Rus & Tolley, 2015; Shih et al., 2020; Yasa et al., 2023). Artificial muscles, emulating biological force generation, are key to innovations in musculoskeletal robotics and assistive technologies (Mirvakili & Hunter, 2018; Li et al., 2022).*

We also added new references in the second Introduction paragraph regarding the modeling and control challenges as follows:

> *These effects lead to memory, hysteresis, and other nonlinearities that complicate analysis and real-time control (Yasa et al., 2023; Della Santina et al., 2023).*
>
> *Under these conditions, analytic ROMs become limited and hard to scale, motivating a shift toward data-driven modeling that captures system dynamics directly from data without precise parameter identification (Yasa et al., 2023; Della Santina et al., 2023).*

In the Discussion, we added:

> *Recent soft-robotics reviews similarly identify accurate but tractable modeling, control of nonlinear compliant dynamics, and deployment robustness as central barriers for fielded soft robotic systems (Yasa et al., 2023; Della Santina et al., 2023; Li et al., 2022).*

(2) **I am confused on why the paragraph starting in Line 256 is not under section 2.3. Since it discusses results for the analytical HASEL model?**

The Reviewer is correct. The paragraph mentioned by the Reviewer was indeed referring to the previous section. We have therefore moved it to the end of Section 2.3.2.

(3) **Line 271: Is the antagonistic arm setup similar to that described by Kazemipour et al. [26], or are there any differences? The sentence in line 271 appears to be inspired, and I'm curious if it's been further developed. Could you please explain the modifications made to the setup?**

The antagonistic joint follows the same conceptual architecture as Kazemipour et al.: two HASEL muscles acting through electrostatic clutches, one side transmitting force while the other is released. It is not, however, a direct reuse of that hardware. We modified the HASEL actuator geometry, the link lengths/lever arms, and the clutch geometry. The clutch change primarily affects its operating voltage and holding margin, while the HASEL and link changes alter the joint kinematics, range of motion, and voltage–angle relation; the slow-manifold map g and controller were therefore identified and validated on the present setup.

We clarified this in the Results in Section 2.4:

> *The present hardware is a modified implementation of the same antagonistic HASEL–clutch architecture: the HASEL actuator geometry and link lengths differ from the previous setup, altering the joint kinematics, range of motion, and voltage–angle relation, while the clutch geometry mainly changes the operating voltage and holding margin because the clutch is used here as a binary engagement element. The slow-manifold map and controller are therefore identified and validated on the present setup.*

(4) **Why do authors not show the prediction performance of aSSM for antagonistic muscles in Figure 6d? Authors say in paragraph starting in line 279 that the hysteric behavior of actuators makes it challenging to use aSSM. Why is hysteresis not a problem for a single HASEL actuator and becomes challenging for the antagonistic case? Can they clarify this point?**

We thank the Reviewer for raising this important point. Figure 6d does not display an aSSM prediction for the antagonistic joint because, for this setup, we constructed only the SM model. The aSSM workflow relies on transient decay trajectories generated by step-like voltage changes around fixed input levels. While this protocol can be applied in a controlled way to the isolated single-actuator case, it becomes less suitable for the antagonistic joint, where two HASEL actuators are mechanically coupled through the joint and clutches and where joint friction and clutch-state history can amplify path-dependent hysteretic effects. These transients are not representative of the normal forced-response trajectories used for control. We therefore used the SM model, which can be identified directly from operational forced-response data.

Following the Reviewer's suggestion, we have clarified this point at the end of section 2.4:

> *Unlike isolated actuators, designing decaying trajectories for artificial muscles is more challenging due to the complexity of the multi-input configuration and the mechanical coupling, which introduces friction at the joint. This makes the use of aSSMs less practical, as their construction relies on transient data following step inputs (see Section 2 of the Supplementary Material), which induce undesirable hysteretic behavior that is atypical under normal operating conditions. In contrast, the SM-based approach demonstrates its practicality by requiring only forced trajectories for training.*

(5) **Figure 6d - Are there any uncertainty regions?**

We thank the Reviewer for this comment. We have added uncertainty regions to the relevant Fig. 6 prediction panels. These regions were estimated from repeated actuator characterization measurements. We compared the displacement values measured during increasing- and decreasing-voltage ramps at

the same voltage, and used the largest observed difference at each voltage as the uncertainty width. The shaded region shows the nominal response plus or minus half of this width, giving a maximum displacement band of ±0.8 mm. For the joint-deflection panel, this displacement uncertainty was converted to angular uncertainty using the 4 mm moment arm, giving a maximum angular band of ±11.4°. We have also added this calculation to the Methods section.

We revised the caption of Fig. 6 as follows:

> *(**b**) Predictions of the actuator stroke under random forcing: the aSSM-based prediction (green) quickly converges to the SM-based prediction (orange), both closely matching the test trajectory (black); the shaded region indicates the uncertainty region estimated from repeated HASEL actuator characterization trials.*
>
> *(**d**) Slow-manifold prediction of the joint deflection under random excitation, demonstrating accurate tracking of the test trajectory; the shaded region shows the corresponding angular uncertainty converted using the 4 mm moment arm.*

We also added the following Methods description:

> *The uncertainty region was estimated from repeated HASEL actuator characterization measurements. Displacement responses were compared at uniformly spaced voltage values between 0 and 7 kV, with a spacing of 0.01 kV. At each voltage value, the uncertainty width was defined as the largest difference between the displacement measured during increasing- and decreasing-voltage ramps across the selected trials. The plotted band corresponds to the nominal response ± half of this width. For joint-angle data, displacement uncertainty was converted to angular uncertainty using the 4 mm moment arm.*

(6) **Figure 8a - x-label is missing**

We thank the Reviewer for pointing this out. We corrected Fig. 8a by adding the missing x-axis label.

(7) **Can SM make predictions for angles outside the range tested in this paper? For instance, in Figure 8a, the maximum angle tested is approximately 40 degrees. However, Supplementary Movie 2 demonstrates that the arm can reach angles of up to 90 degrees. Does SM have the capability to extrapolate to these values, or is retraining necessary? Is there a specific reason why large angles, such as those not tested, have not been explored?**

We thank the Reviewer for this important comment. Our model shows generalization beyond the specific trajectory used for training. We tested this by training the SM model on a single trajectory and then evaluating it on another trajectory with a larger maximum angle deflection than the training trajectory, as shown in Fig. 1a. As in the rest of the paper, we approximate the manifold using a 7th-order polynomial, which provides a balance between accuracy and generalizability. Consequently, even though the test trajectory extends beyond the training trajectory's angle range, the model still delivers accurate predictions, as illustrated in Fig. 1b, achieving an $NMTE = 7.5\%$, without requiring retraining.

The approximately 90° motion shown in Supplementary Movie 2 corresponds to a dynamic demonstration on the earlier Kazemipour et al. platform, rather than to the closed-loop tracking experiment of Fig. 8 on the present setup. In the present work, larger controlled excursions were not explored because the Fig. 8 experiments were performed within a conservative calibrated voltage–angle range selected for repeatable, prolonged actuation with sufficient breakdown margin. Controlled tracking over larger angles can be addressed with the same SM workflow by extending the calibration data to the desired operating range and, if needed for faster motions, including rate-dependent terms.

(8) **I believe the rho number, which defines the dimensionless measure of slowness, is a useful metric that authors define. I'm curious to know the rho values for the tests in Figures 6 and 8. If possible, could they provide the rho values for all the experiments presented in this paper?**

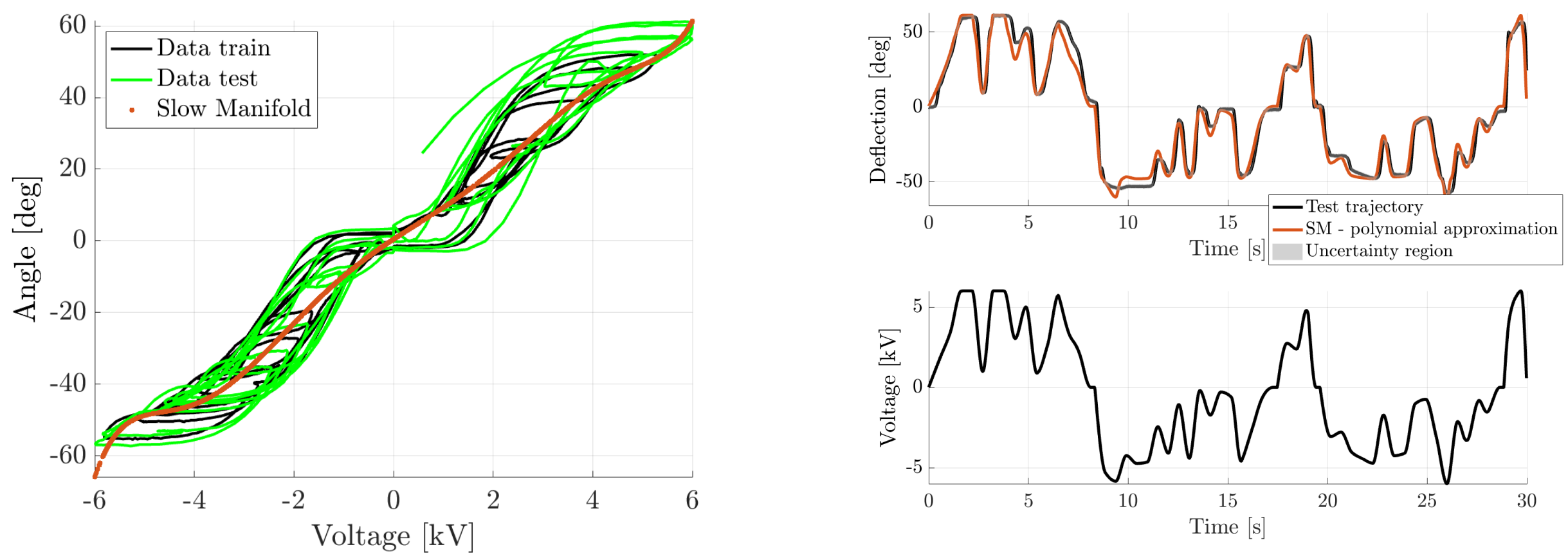

(a) (b)

Figure 1: Generalizability of the slow manifold-based model reduction tested on experimental data for the antagonistic joint system. The model is trained on a single trajectory with a smaller maximum deflection angle than the test trajectory (1a), yet still accurately predicts deflections outside the training range (1b).

Originally, we did not include the value of ρ for the antagonistic joint, as purely decaying trajectories, which are necessary to compute the denominator in the definition of Eq. (7) of the manuscript, were not available. However, we acknowledge the importance of reporting a value of ρ also for the antagonistic joint in order to better understand its meaning, compare it with the isolated HASEL actuator case, and identify a typical value under normal operating conditions. To address this point, we isolated, from the available forced-response data, a portion in which the external forcing rapidly decreases, approximately resembling a step input. This allows us to interpret the corresponding system response as an effective transient decay $s(t)$ in the sense of Eq. (5), thereby enabling the computation of ρ also for the antagonistic joint. While the value of ρ for the isolated HASEL actuator in Fig. 6 was already reported in the text (line 259), following the Reviewer's suggestion, we now additionally report the values of ρ for the antagonistic joint in both Figs. 6 and 8. Specifically, we added the following text at the end of Section 2.4 for the trajectory shown in Fig. 6:

> *As shown in Fig. 6d, the SM-based model accurately predicts the joint deflection with slowness measure $\rho = 0.22$, yielding a normalized mean trajectory error of $\mathrm{NMTE}_{\mathrm{SM}} = 8.13\%$.*

and the following text in Section 2.5 for the trajectory shown in Fig. 8:

> *The test trajectory features a slowness measure of $\rho = 0.15$.*

(9) **Methods, equation 27: what is S_i? Can you define it before and after the equation?**

We thank the Reviewer for pointing this out. We now explicitly define S_i in the manuscript.

$$x_\epsilon(u) \doteq s(u) = \sum_{i=0}^{n} S_i u^i, \quad S_i \in \mathbb{R}, \tag{27}$$

> *where S_i denotes the coefficient of the i-th order term in u.*

(10) **How do authors address the hysteresis of the artificial muscles in their control algorithm and modeling? Are these effects negligible for the conditions considered in this paper? Or are they not applicable to the HASEL robots, despite being mentioned in the abstract? Alternatively, are these effects time-scales that are much faster than the control frequency?**

This is an important point. We would like to emphasize that, in this work, we do not attempt to explicitly model hysteresis, as we assume its effect to be negligible within the operating regime of the artificial muscle. This observation further motivates the use of the SM approach, rather than the aSSM reduction. Indeed, the construction of an aSSM requires transient data generated by large step changes in the applied forcing, which can trigger hysteretic behavior that is atypical of normal operating conditions. Following the Reviewer's comment, we have revised the abstract to clarify how our approach avoids inducing hysteresis during data collection:

> *We learn an explicit input-to-output map on this manifold directly from forced-response trajectories, eliminating the need for decay experiments generated by large step inputs, which can induce pronounced hysteretic behavior and are often unrepresentative of normal operating conditions.*

In our setting, any residual hysteretic effects are not explicitly represented in the reduced-order model. Instead, they are compensated by the feedback controller. Evidence of this can be seen in Fig. 8b, where the *feedback contribution* accounts for discrepancies between the model predictions and the measured system response. We have also made this point more explicit in Section 2.5 by adding the following sentence:

> *This also suggests that the residual hysteretic effects, while small, are effectively handled by the feedback controller.*

(11) **SM Figure 1 presents the experimental data, and I'm curious to know the voltage levels involved. Are these voltages pertinent to the operating condition of the antagonistic arm? Additionally, is there any hysteresis observed within the operational voltage range?**

Referring to Fig. 1b of the Supplementary Material, the HASEL actuator was operated at a baseline voltage of 5 kV, upon which voltage jumps of $\pm[1.5, 2]$ kV were applied to deliberately trigger hysteretic behavior. Following the Reviewer's suggestion, we now explicitly report these voltage levels in the caption of Fig. 1 of the Supplementary Material.

> *Here, we report the actuator driven around a baseline voltage of 5 kV with voltage jumps of* $\pm[1.5, 2]$ *kV.*

For the antagonistic joint, the operating voltage range can reach similar values. However, the voltages are not varied as abruptly as in the experiments of Fig. 1b, where large step changes were intentionally introduced to reveal hysteresis. Consequently, the type of pronounced hysteretic behavior observed in Fig. 1b is not encountered under normal operating conditions of the antagonistic joint. As discussed in our response to the previous comment, any residual hysteretic effects that may still be present in the operating regime are small and are effectively compensated by the feedback controller rather than being explicitly represented in the reduced-order model.

(12) **Supplementary movie 3 appears to be presented at a much faster pace compared to real time. I kindly request reviewers to indicate this in the movie. Similarly, supplementary movie 2 could also benefit from the authors marking whether it is presented in real time or not.**

We thank the Reviewer for this comment. We have now fixed the videos with the corresponding reproduction velocity.

(13) **While running SDOF_SM.m and HASEL_actuator.m "Unrecognized function or variable 'embedCoordinates'."**
While running antagonistic_joint.m I got the following error "Unrecognized function or variable 'finiteTimeDifference'."
Also while running HASEL_simulation.m , I got symbolic toolbox error. I suggest authors to mention which MATLAB toolboxes are required to run the examples.

We thank the Reviewer for pointing out this issue. The package *SSMLearn* should be installed before running the scripts in order to load all relevant functions. We now specify this in the README, along with the additional MATLAB toolboxes required.

Response to Reviewer #2

Leonardo Bettini[1†], Amirhossein Kazemipour[2†], Robert K. Katzschmann[2] and George Haller[1]

[1]Institute for Mechanical Systems, ETH Zurich, Leonhardstrasse 21, 8092 Zurich, Switzerland
[2]Soft Robotics Lab, ETH Zurich, Tannenstrasse 3, 8092 Zurich, Switzerland
† These authors contributed equally to this work

June 9, 2026

We thank Reviewer #2 for their positive and insightful comments. Below, we provide detailed responses to each of the points raised, including excerpts from the revised manuscript with the corresponding changes highlighted in blue.

(1) **Applying data-driven model reduction to soft robotic control is a significant contribution. The modeling and control of soft artificial muscles has been a persistent challenge due to nonlinearities, hysteresis, and multi-physics coupling. Prior work on SSM-based control has been demonstrated in simulation and on continuum soft robot, but this paper represents an experimental demonstration on antagonistic artificial muscle systems with quantified performance improvements over standard baselines. The distinction between aSSM and SM approaches is well motivated: the SM-based method avoids decay experiments that can trigger hysteresis in HASEL actuators, which is a practical advantage over the aSSM construction. The authors appropriately compare to existing quasi-static models, frequency-domain identification, and Port-Hamiltonian approaches, clearly positioning their contribution as a data-driven alternative that avoids detailed physical parameter identification.**

We thank the Reviewer for this positive assessment of the significance and positioning of the manuscript.

(2) **The authors claim that their SM-based model is "sample-efficient," "predictive," and "computationally light," and that the combined feedforward+feedback controller achieves substantially lower tracking errors. The experimental results in Figures 6 and 8 generally support these claims. The SM model trained on seven forced trajectories achieves single-digit NMTE on both single-actuator and antagonistic-muscle tests, supporting the sample-efficiency claim. The control comparison in Figure 8 clearly demonstrates the performance advantage of the combined controller over the baselines. However, the claim that this approach is broadly applicable to "other soft robotic systems that exhibit pronounced time-scale separation" (e.g., PAMs, DEAs, SMA actuators) is not supported by any evidence beyond HASEL actuators. This is a speculative statement and should either be toned down or supported with at least a brief numerical demonstration on a different actuator class. The paper claims in the abstract that the approach "avoids decay experiments that can trigger hysteresis," but the aSSM model (which does use decay data) is also presented extensively throughout the results. The narrative would benefit from a clearer, earlier distinction between the aSSM and SM workflows and when each is appropriate, so that the reader does not come away confused about whether decay data is needed or not.**

We thank the Reviewer for raising these important points. We agree that the broader applicability should be stated as a conditional extension rather than as a demonstrated result for all soft-actuator classes. We therefore revised the Discussion to clarify that our experimental validation is on HASEL-based artificial muscles, and that extension to other soft robotic systems requires the same experimentally verifiable ingredients used here.

> *The demonstrated approach may also be relevant beyond HASEL actuators. Other soft robotic systems, such as pneumatic artificial muscles (PAMs), dielectric elastomer actuators (DEAs), or shape-memory alloy (SMA) actuators, can exhibit internal transients that are fast relative to the commanded motion. When such time-scale separation is experimentally verified, the proposed workflow may provide a sample-efficient route to reduced-order modeling.*

The Reviewer also raises an important point regarding the distinction between the aSSM and SM workflows. The central contribution of this work is the direct reduction onto the SM, which can be constructed exclusively from forced-response trajectories. Unlike the aSSM procedure, this approach does not require transient decay experiments generated by large step inputs, which may induce pronounced hysteretic behavior and are often unrepresentative of normal operating conditions. To make this distinction clearer, we have revised the abstract as follows:

> *In the adiabatic regime, where inputs vary slowly relative to intrinsic transients, trajectories rapidly converge to a low-dimensional slow manifold. We learn an explicit input-to-output map on this manifold directly from forced-response trajectories, eliminating the need for decay experiments generated by large step inputs, which can induce pronounced hysteretic behavior and are often unrepresentative of normal operating conditions.*

Following the Reviewer's suggestion, we have also clarified throughout the manuscript that the primary focus of the paper is the SM-based reduction rather than the aSSM procedure. While a detailed presentation of SSM theory remains necessary to introduce the slow-manifold formulation, the role of the aSSM in this work is primarily comparative; it serves to demonstrate that the proposed SM approach achieves essentially the same predictive accuracy while avoiding the transient decay experiments required for aSSM construction. To emphasize this point, we added the following text to Section 2.4:

> *For comparison, we also construct an aSSM-based model using a cubic-order approximation for both the manifold parametrization and the reduced dynamics. As shown in Fig. 6b, the prediction from the aSSM model rapidly converges to that of the SM model, offering marginal improvement. This demonstrates that the simpler SM approach, trained solely on forced trajectories, can achieve essentially the same predictive performance without requiring transient decay experiments.*

(3) **I have not found significant errors in the data analysis or interpretation. The NMTE metric is well-defined and consistently applied. The control comparison is conducted under identical safety constraints and clutch scheduling, which is a fair benchmarking protocol. One concern is that the PI gains were tuned separately for the combined and feedback-only controllers. While the authors justify this for fairness and stability, it makes it difficult to isolate the contribution of the feedforward term. A supplementary experiment using the same PI gains in both the feedback-only and combined conditions would strengthen the comparison.**

The combined controller in Fig. 8 already operates at no stronger PI feedback than PI-only: it uses the same integral gain ($k_i = 0.12$) and a lower proportional gain ($k_p = 0.04$ vs. 0.08). Under these gains, FF+PI achieved RMS error $2.38°$ and peak absolute error $10.41°$, compared with $7.63°$ RMS and $26.00°$ peak for PI-only.

For completeness, a separate FF+PI trial using the same reference trajectory and the PI-only gain setting ($k_p = 0.08$, $k_i = 0.12$) yielded RMS error $2.06°$ and peak absolute error $10.35°$ (Fig. 1). The

two FF+PI configurations produced similar errors: RMS 2.38° vs. 2.06°, and peak 10.41° vs. 10.35°. The Fig. 8 gains reflect the calibration-trajectory tuning protocol, whereas the matched-gain trial serves as a sensitivity check using the PI-only gains. Together, these two observations indicate that the tracking improvement of FF+PI over PI-only is attributable to the added feedforward term rather than to differences in feedback tuning.

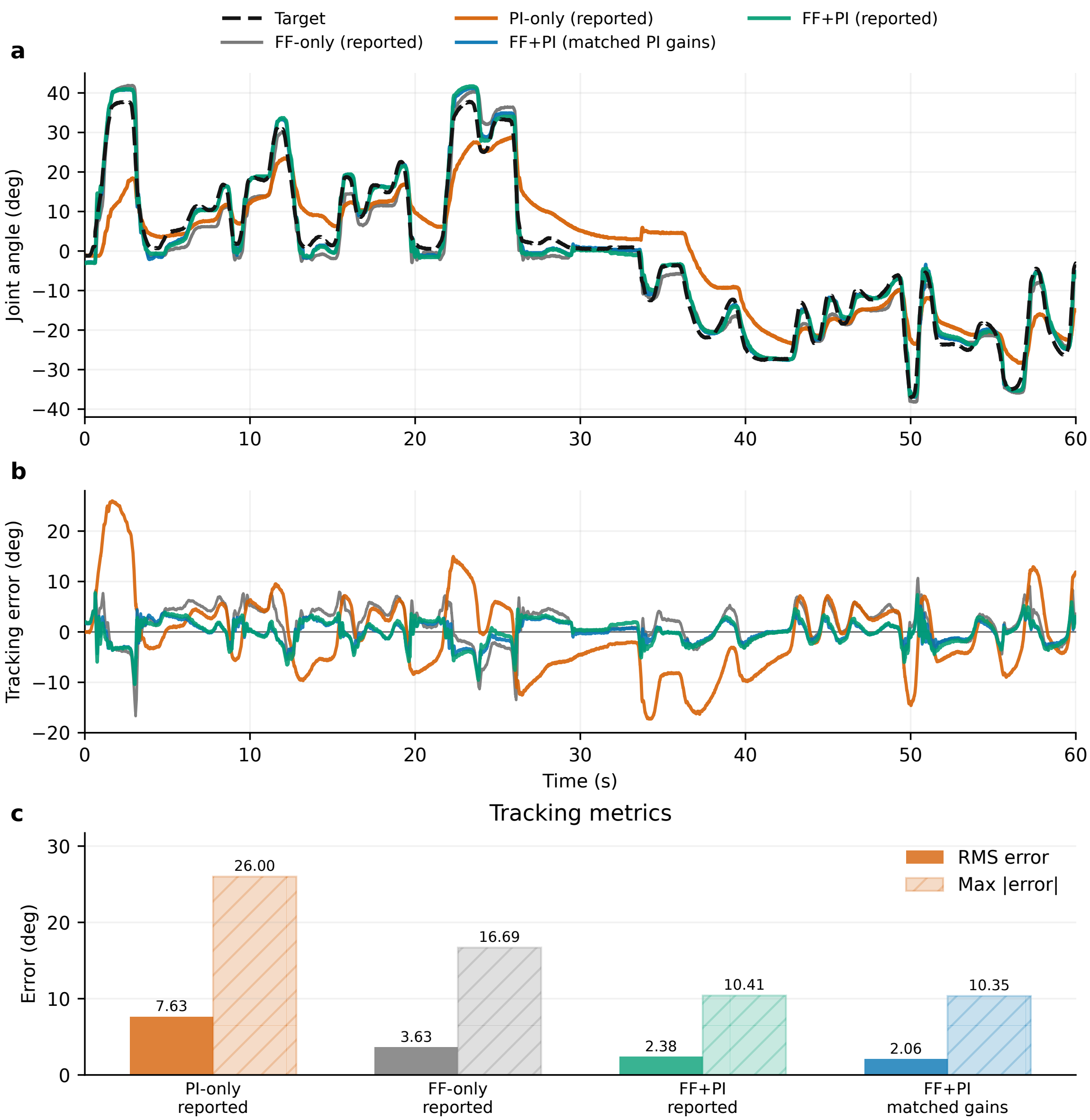

Figure 1: Tracking comparison under matched and unmatched feedback gains. PI-only baseline ($k_p = 0.08$, $k_i = 0.12$); the Fig. 8 FF+PI controller ($k_p = 0.04$, $k_i = 0.12$, both gains component-wise no greater than PI-only); and an additional FF+PI trial using the PI-only gain setting ($k_p = 0.08$, $k_i = 0.12$). Both FF+PI cases retain substantially lower tracking error than PI-only, supporting that the performance improvement arises from the feedforward term rather than from stronger feedback gains.

Following the Reviewer's suggestion, we clarified in the Results section that the reported FF+PI controller used the same integral gain and a lower proportional gain than the PI-only controller. We

also revised the Methods benchmarking protocol to state that PI gains were tuned on a short calibration trajectory and then held fixed for the benchmark trajectory. The matched-gain trial is included here as a sensitivity check using the PI-only gains.

> *The selected gains are* $(k_p, k_i) = (0.08, 0.12)$ *for PI-only and* $(0.04, 0.12)$ *for the combined controller. All controllers are then evaluated on the same randomized test reference trajectory under identical safety constraints and clutch scheduling.*
>
> *Feedforward-only has no PI gains; for the PI-only and combined conditions, PI gains were tuned separately on a short calibration trajectory, targeting low RMS tracking error with stable operation under the same saturation and slew-rate limits, and were then held fixed for the benchmark trajectory.*

(4) **The methodology is generally sound and well-grounded in existing SSM theory. The progression from analytic models to experimental single actuators to the full antagonistic joint is logical and builds confidence in the approach. I have two suggestions regarding the experimental validation: The robustness of the SM model to environmental changes (e.g., temperature variation, actuator aging, payload changes) is not explored. It would be helpful if the authors could comment on or test how the learned polynomial map degrades over time or under different operating conditions, and how frequently re-calibration might be needed. The control experiments are demonstrated on a single randomized test trajectory. Repeating the comparison across multiple distinct test trajectories with varying frequency content and amplitude ranges would provide more convincing statistical evidence of the performance gains.**

We agree that these points are important for interpreting the validation. We distinguish here between hardware-level environmental qualification and controller repeatability on the calibrated experimental setup. The learned map g should not be interpreted as hardware-independent; it is a calibration of the tested antagonistic joint. We have clarified these points in the Discussion:

> *The learned map* g *should be interpreted as a device-specific calibration of the tested antagonistic joint. Hardware and payload changes or long-term aging can shift the voltage–angle relation; in practice, the PI term compensates moderate mismatch, while persistent increases in tracking error or feedback effort indicate the need for recalibration.*

Within this scope, we added an analysis of repeated control data collected on the same physical setup (Fig. 2 and Table 1). The original Fig. 8 trajectory remains the complete three-controller comparison. On the Fig. 8 benchmark trajectory, five FF+PI trials remained in a narrow low-error range, with RMS error $2.35 \pm 0.18°$, compared with $3.63°$ for FF-only and $7.63°$ for PI-only. We also evaluated a second random trajectory with larger target range and higher reference total-variation rate, where FF+PI reduced RMS error relative to FF-only ($5.36 \pm 0.27°$ vs. $6.50°$). Finally, four repeated FF+PI trials on a sinusoidal trajectory gave RMS error $2.42 \pm 0.35°$, showing repeatable performance on a distinct smooth reference class.

Following the Reviewer's suggestion, we added the following Results pointer at the end of Section 2.5:

> *We provide additional repeatability and trajectory-generalization analyses on the same physical setup in Supplementary Fig. 3 and Supplementary Table 1.*

and the following text in Section 4 of the Supplementary Material:

> *We analyzed repeated control data collected on the same physical antagonistic HASEL–clutch setup to assess repeatability of the learned-map-based controller beyond the representative Fig. 8 trial. The original Fig. 8 benchmark trajectory remains the complete three-controller comparison. On this benchmark trajectory, five trials of the combined inverse-SSM+PI controller, denoted FF+PI, including the Fig. 8 configuration and an additional gain-sensitivity trial using the PI-only gain setting, remained in a narrow low-error range. We also analyzed a second random trajectory with a larger target range and higher reference total-variation*

rate, defined as $T^{-1}\int_0^T |\dot{\theta}_d(t)|\,dt$, where T is the trajectory duration and $\theta_d(t)$ is the desired joint-angle trajectory. For sampled data at times t_i, this quantity was computed as $T^{-1}\sum_i |\theta_d(t_{i+1}) - \theta_d(t_i)|$. On this second random trajectory, FF+PI was compared with FF-only. We also evaluated a sinusoidal trajectory, where repeated FF+PI trials tested performance on a distinct smooth reference class. Table 1 reports RMS error and 95th-percentile absolute error because isolated clutch-switching transients can dominate maximum absolute error without representing sustained tracking performance.

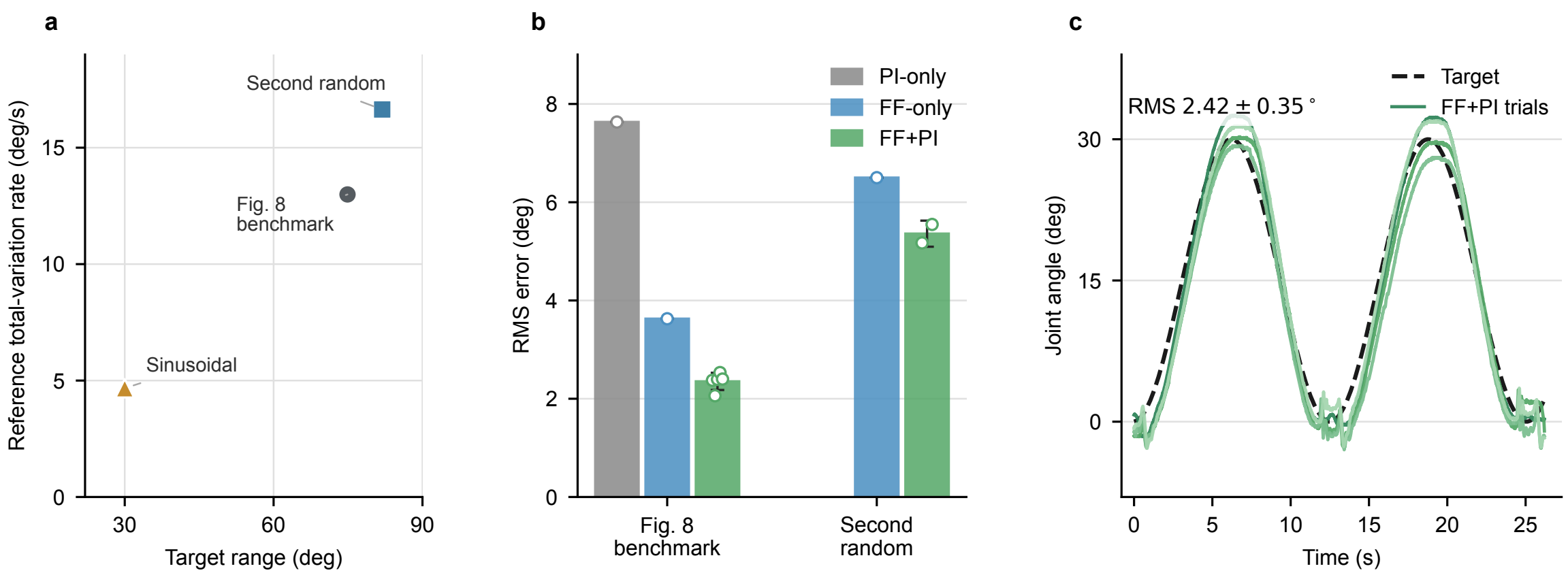

Figure 2: Repeatability and trajectory-generalization analysis. (a) Target range and reference total-variation rate for the Fig. 8 benchmark, second random, and sinusoidal trajectories. (b) Randomized-trajectory comparisons using RMS tracking error. Bars show mean values, error bars show standard deviation for repeated trials, and circles show individual trials. (c) Repeated FF+PI tracking on the sinusoidal trajectory, showing consistent performance of the learned-map-based controller on a distinct smooth reference class. The corresponding 95th-percentile absolute errors are reported in Table 1.

Table 1: Tracking metrics for the additional repeatability and trajectory-generalization analysis. Values are mean ± standard deviation for repeated trials.

Trajectory	Controller	n	RMS error (°)	95th-percentile $\lvert e \rvert$ (°)
Fig. 8 benchmark	PI-only	1	7.63	15.15
Fig. 8 benchmark	FF-only	1	3.63	6.64
Fig. 8 benchmark	FF+PI	5	2.35 ± 0.18	4.21 ± 0.33
Second random	FF-only	1	6.50	8.81
Second random	FF+PI	2	5.36 ± 0.27	5.90 ± 0.47
Sinusoidal	FF+PI	4	2.42 ± 0.35	4.37 ± 0.70

(5) **The authors have provided sufficient detail in the Methods section, and the code and data are made available through an external repository. The step-by-step procedure for data-driven aSSM construction (Methods 4.1) and the SM polynomial fitting (Methods 4.2) are clearly described. The control implementation details, including the anti-windup scheme, clutch scheduling state machine, and safety constraints, are thorough. I am confident that the modeling and control pipeline could be reproduced from the provided information.**

We thank the Reviewer for this positive assessment of the reproducibility of the Methods and control implementation. To further support reproducibility, we have also slightly modified the README document, reporting the necessary toolboxes for running the code.

(6) **The slowness metric ρ defined in Eq. (7) is central to the validity of the SM approximation, yet the paper does not provide clear practical guidance on what threshold of ρ is acceptable for reliable SM-based control. The analytic examples show that $\rho = 0.1$ works well and $\rho = 1.2$ does not, but the experimental HASEL system operates at $\rho = 0.28$. A more systematic study or clearer guidelines on the acceptable range of ρ would make the method more accessible to practitioners.**

We thank the Reviewer for raising this important point. Following the Reviewer's suggestion, we have clarified the practical interpretation of the slowness measure ρ. In the present setting, $\rho < 1$ corresponds to the adiabatic regime in which the external forcing varies more slowly than the intrinsic transient relaxation, and the slow-manifold approximation improves as ρ becomes increasingly smaller than one. This interpretation is consistent with both the analytical and experimental results: as the Reviewer points out, the analytical examples show accurate agreement for $\rho = 0.1$ and clear degradation for $\rho = 1.2$, while the experimental results show accurate predictions for the isolated HASEL actuator at $\rho = 0.28$ and for the antagonistic joint at $\rho = 0.22$ and $\rho = 0.15$. We therefore added the following practical guideline at the end of Section 2.2:

> *In general, we expect the slow-manifold reduction to remain valid for $\rho < 1$, with the quality of the approximation improving as ρ becomes increasingly smaller than one.*

To further support this evidence, we also now report the slowness measure for the antagonistic joint. For the trajectory shown in Fig. 6, we added the following text at the end of Section 2.4:

> *As shown in Fig. 6d, the SM-based model accurately predicts the joint deflection with slowness measure $\rho = 0.22$, yielding a normalized mean trajectory error of* $\mathrm{NMTE}_{\mathrm{SM}} =$ *8.13%.*

For the closed-loop trajectory shown in Fig. 8, we added the following text in Section 2.5:

> *The test trajectory features a slowness measure of $\rho = 0.15$.*

(7) **The assumption that only one side of the antagonistic muscle is active at a time (mutually exclusive clutch engagement) simplifies the input to a single scalar. The authors acknowledge this as a limitation but do not discuss how significantly this restricts the operational envelope of the joint. It would be useful to discuss in what practical scenarios bilateral co-contraction (simultaneous engagement) would be needed and how much performance is sacrificed by excluding it.**

We agree that the mutually exclusive clutch schedule defines the operating mode of the present controller. The resulting scalar-input SM is intended for position tracking under alternating antagonistic actuation.

The main behavior excluded by this choice is bilateral co-contraction. Such operation is relevant when the task requires independent stiffness or impedance modulation, bidirectional load holding, disturbance rejection, or preload during rapid reversals. These cases are outside the present alternating-actuation benchmark and would require a higher-dimensional SM map with independent left/right actuator and clutch inputs.

Following the Reviewer's suggestion, we clarified this point at the end of Section 2.4:

> *This scalar-input representation specializes the antagonistic joint to position tracking under alternating actuation; co-contraction commands for joint-impedance modulation are discussed as a future extension in the Discussion.*

and in the Discussion:

> *This scalar-input specialization restricts the controller to alternating-actuation position tracking. Tasks requiring bilateral co-contraction, such as independent stiffness or impedance modulation, bidirectional load holding, disturbance rejection, or preload during rapid reversals, would require a higher-dimensional SM map with independent left/right actuator and clutch inputs.*

Based on these limitations, future work will target (i) asynchronous HASEL/clutch activation and simultaneous bilateral actuation via higher-dimensional aSSMs/SMs, enabling tunable joint stiffness for impedance-modulated tasks; (ii) hysteresis-aware or rate-dependent corrections for faster inputs; (iii) online adaptation of g to compensate drift; (iv) principled optimization of clutch timing to balance performance and safety; and (v) in-loop fault detection for HASEL dielectric breakdown and clutch slip to enable safe field deployment.

(8) **In Figure 6d, the SM prediction of joint deflection shows noticeable deviations at the peaks and sign transitions of the trajectory. It would be helpful if the authors could discuss whether these errors are due to the clutch switching transients, hysteresis effects, or limitations of the polynomial approximation order.**

It is indeed true that the prediction in Fig. 6d exhibits slight deviations from the measured trajectory, particularly near some extrema and sign transitions. As the Reviewer suggests, they may arise from residual hysteretic effects, limitations of the polynomial approximation of the slow manifold and reduced dynamics, or the complexity of the antagonistic joint system itself, including clutch engagement and disengagement events.

To better assess the significance of these discrepancies, we have now included the experimental uncertainty region in Fig. 6d, reported below. As seen, although the prediction exhibits small deviations from the measured trajectory, it remains largely within the uncertainty bounds of the experiment. This suggests that the observed discrepancies are comparable to the overall variability introduced by the experimental setup and the complexity of the system. Consequently, they do not significantly affect the predictive capability of the reduced-order model.

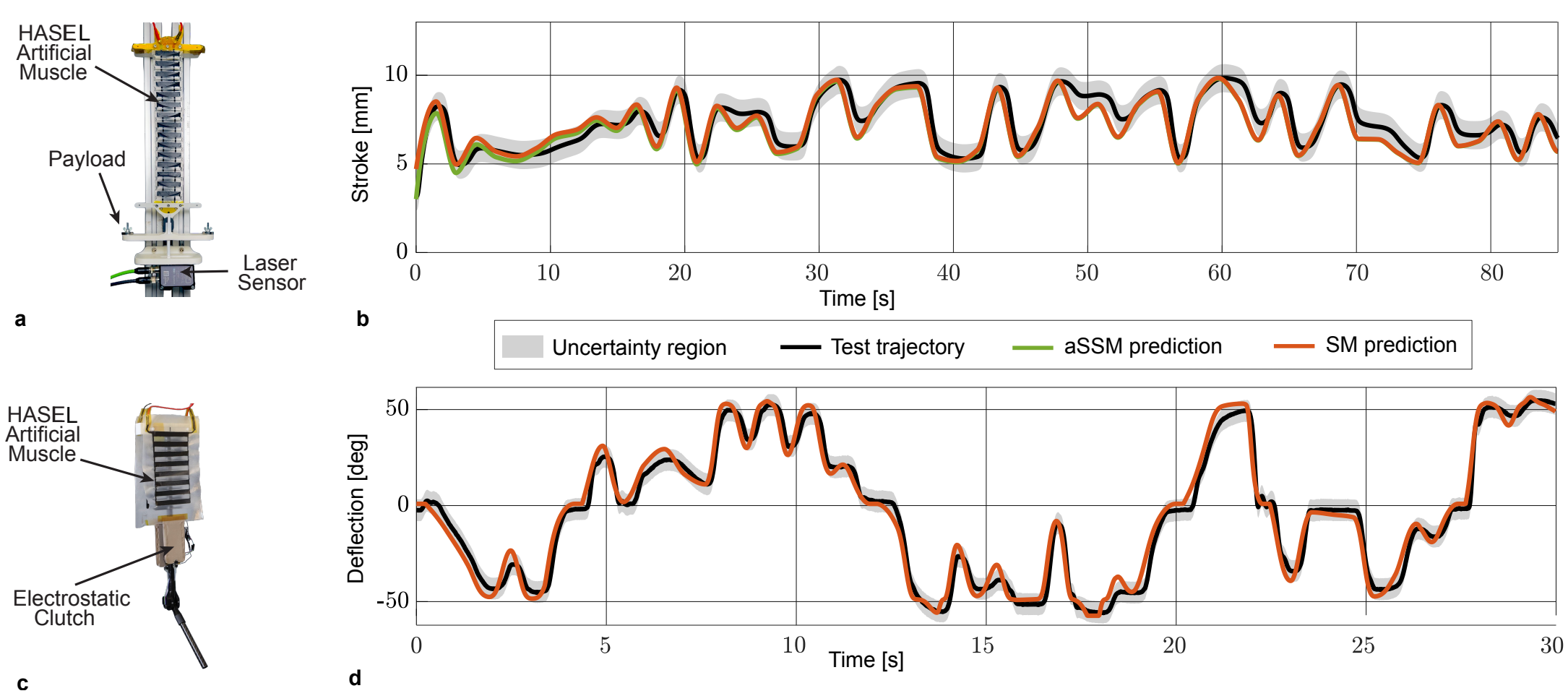

Figure 6: ***Experimental HASEL artificial muscles and data-driven slow manifold predictions.*** *(**a**) Single HASEL actuator with payload and laser displacement sensor. (**b**) Predictions of the actuator stroke under random forcing: the aSSM-based prediction (green) quickly converges to the SM-based prediction (orange), both closely matching the test trajectory (black); the shaded region indicates the uncertainty region estimated from repeated HASEL actuator characterization trials. (**c**) Musculoskeletal joint with an antagonistic pair of HASEL actuators and electrostatic clutches. (**d**) Slow-manifold prediction of the joint deflection under random excitation, demonstrating accurate tracking of the test trajectory; the shaded region shows the corresponding angular uncertainty converted using the 4 mm moment arm.*

(9) **The paper would benefit from a brief discussion of failure modes. Under what conditions would the SM-based controller produce unsafe or unstable behavior? For instance, what happens if the actuator experiences dielectric breakdown or if the clutch fails to engage?**

We thank the Reviewer for raising this important point. We added a Discussion paragraph distinguishing controller-level limits, which are bounded by the safety layers described in Methods, from hardware faults such as HASEL dielectric breakdown and clutch slip, which require sensing or hardware intervention rather than controller-level correction:

> *Beyond gradual drift, failure modes separate into controller-level limits and hardware faults. The safety layers described in Methods keep commands within the configured operating envelope, so out-of-range references or violations of the slow-input assumption lead to degraded tracking or saturation rather than unbounded command growth. Hardware faults, such as HASEL dielectric breakdown or loss of clutch engagement, fall outside the controller's corrective authority and would require actuator replacement or fault detection, for example through voltage/current monitoring or clutch slip sensing.*
